# Is there a role for patients and their relatives in escalating clinical deterioration in hospital? A systematic review

**DOI:** 10.1111/hex.12496

**Published:** 2016-10-26

**Authors:** Abigail K. Albutt, Jane K. O'Hara, Mark T. Conner, Stephen J. Fletcher, Rebecca J. Lawton

**Affiliations:** ^1^ School of Psychology University of Leeds Leeds UK; ^2^ School of Medicine University of Leeds Leeds UK; ^3^ The Mid Yorkshire Hospitals NHS Trust Wakefield, UK; ^4^ Bradford Institute for Health Research Bradford Royal Infirmary Bradford UK

**Keywords:** critical care, patient safety, patient‐centred care, rapid response team, systematic review

## Abstract

**Background:**

Measures exist to improve early recognition of, and response to, deteriorating patients in hospital. However, deteriorating patients continue to go unrecognized. To address this, interventions have been developed that invite patients and relatives to escalate patient deterioration to a rapid response team (RRT).

**Objective:**

To systematically review articles that describe these interventions and investigate their effectiveness at reducing preventable deterioration.

**Search strategy:**

Following PRISMA guidelines, four electronic databases and two web search engines were searched to identify literature investigating patient and relative led escalation.

**Inclusion criteria:**

Articles investigating the implementation or use of systems involving patients and relatives in the detection of clinical patient deterioration and escalation of patient care to address any clinical or non‐clinical outcomes were included. Articles’ eligibility was validated by a second reviewer (20%).

**Data extraction:**

Data were extracted according to pre‐defined criteria.

**Data synthesis:**

Narrative synthesis was applied to included studies.

**Main results:**

Nine empirical studies and 36 grey literature articles were included in the review. Limited studies were conducted to establish the clinical effectiveness of patient and relative led escalation. Instead, studies investigated the impact of this intervention on health‐care staff and available resources. Although appropriate, this reflects the infancy of research in this area. Patients and relatives did not overwhelm resources by activating the RRT. However, they did activate it to address concerns unrelated to patient deterioration.

**Conclusions:**

Activating a RRT may not be the most appropriate or cost‐effective method of resolving non‐life‐threatening concerns.

## 1 BACKGROUND

Clinical deterioration is marked by a period of clinical instability^1^ which can occur at any time during a patient's illness, but is more common following emergency admission to hospital, after surgery and during recovery from a critical illness.^2^ In‐hospital clinical deterioration that is not promptly responded to can lead to numerous severe consequences for the patient including increased length of hospital stay, cardiac arrest, admission to the intensive care unit (ICU) and increased morbidity and mortality.^3^, ^4^, ^5^ Such serious adverse events may be prevented by recognizing and responding to early signs of clinical deterioration.^6^, ^7^

To aid the recognition of and response to clinical deterioration, early warning score (EWS) systems and rapid response teams (RRT) have been introduced in countries including the UK, USA and Australia.^8^ EWS is based on routine physiological measurement of patients’ vital signs from which a score is calculated and recorded. When a patient's EWS is outside the normal range, this can be indicative of clinical deterioration and can prompt health‐care staff to escalate patient care and trigger a RRT. A RRT typically consist of medical and nursing staff with critical care skills that provide timely treatment to support the deteriorating patient on the ward.^9^ However, evidence for the efficacy of EWS and RRT systems at reducing in‐hospital mortality is equivocal.^10^, ^11^, ^12^ The management of critical illness remains a problem as some patients who are deteriorating continue to go unrecognized and appropriate, timely action is not always taken.^13^

Increasingly, patients are empowered to be active partners in their health care, with treatment decisions ideally being made between health‐care staff, patients and their relatives^14^, ^15^ and patient involvement is promoted as a means of improving patient safety.^14^, ^16^ Detecting clinical deterioration and escalating care is one such area where patients and their relatives could be involved. Nurses may identify patient deterioration using intuitive reasoning that develops with experience^17^ and that is mediated by their knowledge of the patient.^8^ It is intuitive to think that patients and their relatives have knowledge of the patient and their norms, and may sense whether the patient's clinical condition is deteriorating. This has been especially well documented in paediatric deterioration where relatives’ recognize signs that the patient is deteriorating before health‐care staff.^18^

There is a growing acceptance of patient and relative led escalation in health‐care services, and it has been implemented in a number of institutions. Indeed, a recent study that aimed to determine the prevalence and characteristics of RRTs in hospitals in the USA found that 69% of 103 institutions had introduced patient and relative led escalation.^19^ Therefore, it is important to understand how patients and their relatives recognize and escalate deterioration using these systems, and whether these systems are effective at preventing deterioration, to indicate how patients and their relatives can contribute towards improving the management of clinical deterioration in hospital. This article aimed to systematically review citations that: (i) identify and describe systems involving patients and relatives in the process of escalating in‐hospital clinical deterioration, (ii) describe how these systems have been implemented, and (iii) investigate the effectiveness of these systems at preventing in‐hospital clinical deterioration. This topic will be summarized with regard to the available peer reviewed, academic literature and non‐peer reviewed, grey literature. A decision was made to include grey literature to examine what is happening in practice, and also because practitioners may not have the same incentive as academics to publish in peer‐reviewed journals.^20^ The implications of engaging patients and relatives in the escalation of clinical deterioration will also be outlined.

## 2 METHODS

### 2.1 Search strategy

This systematic review was guided by the Preferred Reporting Items for Systematic Reviews and Meta‐analyses statement (see Supplementary material 1), and the protocol was published on PROSPERO (Registration number: CRD42015019246). Search terms used included combinations of “patient, family OR relative activated” AND “rapid response team, medical emergency team, critical care outreach OR condition help” AND “patient deterioration.” The search strategy was applied to PsycINFO, MEDLINE, Cumulative Index to Nursing and Allied Health Literature (CINAHL) and Cochrane Library in February 2015. Searches were limited to retrieve articles published in the years following 1990. This time restriction was used because RRT was first developed around this time.^21^

To identify grey literature, web search engines (Google and Google Scholar) were selected and searched. The web search engines could not accommodate the full search strategy used in the electronic databases. Therefore, a simpler search strategy was used in the web search engines. Terms were searched for in the titles of pages and anywhere else in the text. This search strategy produced predictably large numbers of results. Subsequently, the first 100 results of each grey literature search in Google and Google Scholar were reviewed for relevance.^22^ The academic and grey literature search strategies and full results are detailed in Supplementary material 2.

### 2.2 Eligibility criteria and study selection

Eligibility criteria applied to academic literature are defined in Table 1. For grey literature, eligibility criteria used were the same as that applied to academic literature except it was not necessary for grey literature to use comparison groups or outcome measures. The titles and abstracts of identified citations were screened against the inclusion criteria, and the full texts of potentially relevant citations were obtained and reviewed for inclusion by one reviewer (AA). A random sample of 20% of the citation titles and abstracts was screened independently against inclusion criteria by three‐second reviewers (RL, JOH, MC). To resolve any discrepancies in citation inclusion, a discussion was held between the reviewers to reach a consensus. After discussion, the eligibility criteria for grey literature were altered. When screening citations against the new grey literature eligibility criteria, 100% consensus was reached for citation inclusion.

**Table 1:** Eligibility criteria for the inclusion of academic articles in the review

PICOS	Eligibility criteria
Population	Adult and paediatric patients hospitalized in developed countries and their relatives or carers.
Intervention	Implementation or use of systems involving patients and relatives in the detection of clinical patient deterioration and escalation of patient care.
	Systems implemented alone or within a complex intervention.
Comparison	Detection and escalation by patients and relatives can be compared to detection and escalation by any other group.
Outcome	Patient and relative detection and escalation could be used to address any clinical and non‐clinical outcome.
Study design	Peer‐reviewed reports of empirical, academic research were included. Non‐peer‐reviewed articles and grey literature were also included. Opinion pieces were excluded.
	Articles using any study design, published after the year 1990 were included.
	Only studies published in English were included because of limited resources for translation.

### 2.3 Assessment of study quality

Study quality was assessed using the Quality Assessment Tool for Studies with Diverse Designs (QATSDD).^23^ The QATSDD is a validated quality assessment tool, comprising of 14 items on a four‐point scale that can be applied to a methodologically diverse group of studies. The studies were scored to indicate the quality of the individual studies and the overall scope of research. One reviewer conducted quality assessments for all studies (AA), and then, three reviewers conducted a second quality assessment of all studies (RL, JOH, MC). There was a strong, significant correlation between the first and second reviewers’ quality assessments, *r*=.73, *P*=.039.

### 2.4 Data extraction and synthesis

Data were extracted according to pre‐defined criteria by a single reviewer (AA) for citations accepted after full text screening (see Supplementary material 3 for the data extraction form). The accuracy and completeness of data extraction was independently assessed by three‐second reviewers (RL, JOH, MC). Owing to the heterogeneous designs of the included studies, narrative data synthesis was carried out on the academic and grey literature using guidance from Popay et al.^24^ Narrative data synthesis is an approach in which the findings from multiple studies are summarized and synthesized principally using words^24^ as opposed to numbers. Preliminary descriptions of the results of each of the citations were developed using textual descriptions, categories and tabulations, and then, an understanding of the relationship between individual study characteristics and their findings was explored.^24^ The categories so derived are presented under subheadings in the results section.

## 3 RESULTS

**Figure 1:**
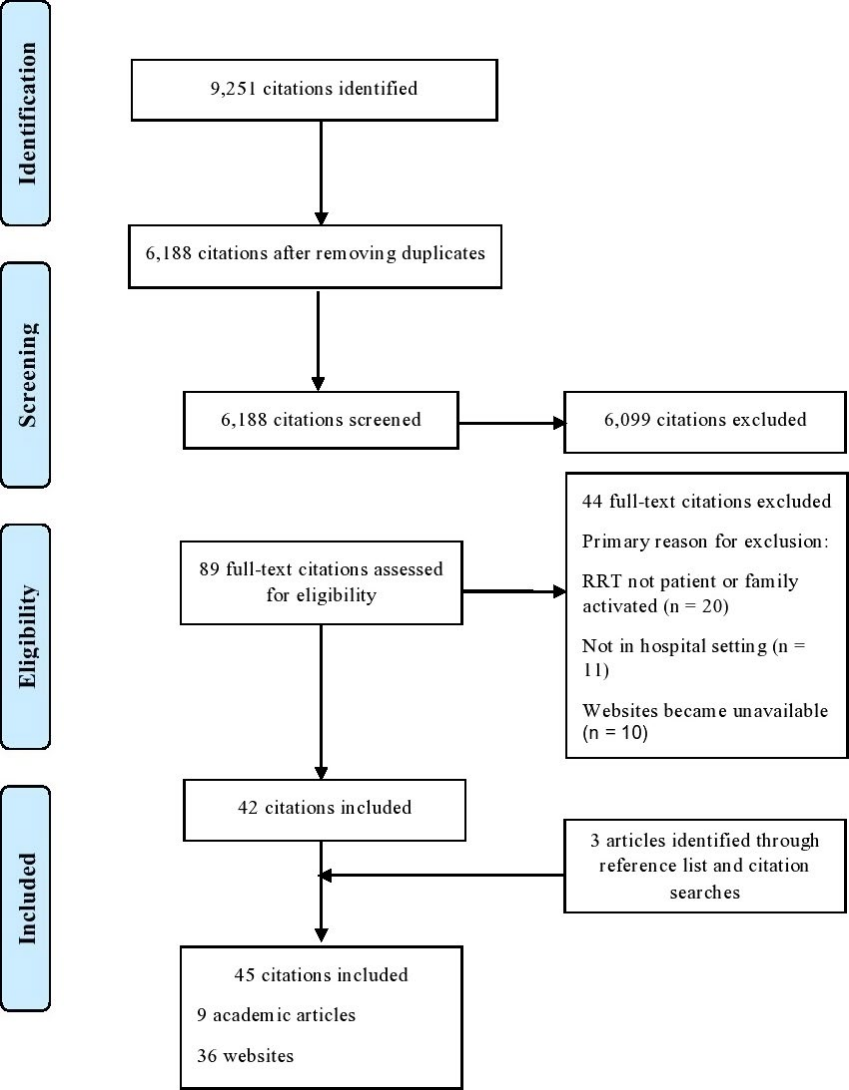
The PRISMA flow diagram details our search and selection process and includes the number of citations identified, included and excluded, and the reasons for exclusion

A total of 6188 potential citations were identified after de‐duplication. After title and abstract screening, 89 citations potentially fulfilled the eligibility criteria. The full texts of these citations were acquired and reviewed. Of these, nine academic articles from the academic literature search and 36 websites from the grey literature search fulfilled the eligibility criteria and were included in the review (Fig. 1). The key characteristics of included citations are outlined in Supplementary material 4. Of the academic articles, patient and relative led escalation was most often researched in the USA, within single centres (eight of nine articles), and to address paediatric deterioration (six of nine articles). All nine studies measured at least one non‐clinical outcome. These were the number of patient and relative activated RRT and the reasons for activation,^14^, ^25^, ^26^, ^27^, ^28^, ^29^ number of RRT activations where family concern was noted,^30^, ^31^ percentage of patients and relatives who received education about the service,^27^, ^28^ and a survey to test patient and family understanding,^27^, ^28^, ^29^ and staff understanding.^29^ Three studies also measured clinical outcomes: transfer of the patient to higher level care after RRT assessment,^30^, ^32^ number of non‐ICU adverse events (AE)^32^ and mean number of days between cardiac arrests^31^ since the introduction of patient and relative activated RRT. In terms of the grey literature, the majority of websites were written in the USA. The websites’ target audiences were most often patients and relatives. The majority of websites informed patients and relatives about the origins and purpose of patient and relative led escalation and explained how they can activate a RRT at a particular health‐care organization.

### 3.1 Role of patients and their relatives

The reviewed citations indicate that implemented systems centre on enabling patients and relatives to escalate care for suspected clinical deterioration, placing little focus on how patients and relatives might detect deterioration. While the aims of these systems are consistent, to summon health‐care professionals to assess the patient's clinical condition and treatment needs in a timely manner, institutions appear to subscribe to different patient and relative led escalation protocols and invite different patient groups to engage in this service.

#### 3.1.1 Direct or indirect escalation of care

In five studies, the health‐care organization implemented an indirect pathway of patient and relative led escalation, referred to as Condition Help.^14^, ^25^, ^26^, ^27^, ^28^ Here, patients and relatives activated a Condition Help team which had distinct staff members from the RRT. The Condition Help team triaged the patient to determine whether the RRT was required. In this way, patients and relatives indirectly escalated care through the Condition Help team. In four studies, the health‐care organization implemented a direct pathway of patient and relative led escalation.^29^, ^30^, ^31^, ^32^ With this patient and relative led escalation protocol, the same RRT who respond to clinicians activations could be activated directly by patients and relatives, with no triage step. The indirect pathway, Condition Help, was implemented more often than the direct pathway in the academic literature. This finding is consistent with the included grey literature websites, whereby numerous websites described what Condition Help is, the origins of the service and how the service can be used.

#### 3.1.2 Composition of the RRT

A further distinction identified between studies was the different types and numbers of health‐care professionals used to comprise the Condition Help teams and RRT. This ranged from a nurse, nurse manager, respiratory therapist, resident physician and critical care fellow,^30^ to a respiratory therapist and critical care nurse.^32^ Although different patient and relative led escalation protocols were used, studies exploring patient and relative satisfaction found that they had favourable opinions towards the service.^14^, ^29^, ^32^

#### 3.1.3 Escalation of paediatric or adult deterioration

The academic literature has focused more on investigating patient and relative led escalation for paediatric compared to adult deterioration. Early studies explored the development and implementation of patient and relative led escalation for paediatric deterioration, suggesting that this service was initially available to prevent clinical deterioration in hospitalized children.^26^, ^31^ In line with this, evidence indicates that children's clinical conditions can deteriorate at a faster rate than adults.^33^ However, later studies did investigate patient and relative led escalation with adult patients.

### 3.2 Process of implementation

#### 3.2.1 Education for health‐care staff, patients and relatives

In all reviewed studies, health‐care staff, patients and relatives received education about patient and relative led escalation prior to its implementation. Health‐care staff were frequently educated in group sessions where they received information about what the service was and how to educate patients and relatives so they can use it appropriately. Staff had to demonstrate a certain level of understanding about the service before they could educate patients and relatives about it. The grey literature search revealed that guidance was available for health‐care organizations considering implementing patient and relative led escalation. From the academic literature, it was not clear whether health‐care organizations made use of these guidelines. Patients and relatives were often first informed about the patient and relative led escalation by the admitting nurse using a formalized teaching script. Posters and leaflets were also provided in patients’ rooms to remind them and their relatives of the information they received from the admitting nurse. This was reflected in the grey literature websites where numerous patient and relative facing educational leaflets, posters and videos were identified.

#### 3.2.2 Use of small‐scale pilot studies

Six of the studies reviewed made reference to the use of a small‐scale pilot study where patient and relative led escalation was implemented on a small number of hospital wards for a short time period. During the pilot phase, health‐care staff, patients and relatives provided feedback, including potential barriers to their engaging in the service.^31^ Barriers identified for health‐care staff included concerns that patients and families would summon the RRT for frivolous or non‐emergent reasons. Barriers for patients and relatives were not explicitly stated. Parts of the service were revised prior to whole hospital implementation based on the feedback received.^32^

### 3.3 Effectiveness of patient and relative led escalation

#### 3.3.1 Clinical outcomes

The patient and relative led escalation protocols introduced across studies aimed to summon health‐care professionals to assess the patient's clinical condition and treatment needs in a timely manner, treat patients accordingly and subsequently prevent clinical deterioration. Gerdik et al.^32^ reported a significant increase in transfers to higher level care and a non‐significant decrease in the number of non‐ICU AE when comparing the phase before RRT implementation and the phase after RRT and patient and relative led escalation implementation. Also, Ray et al.^31^ reported an increase in the median number of days between cardiac arrests from 34 days to 104 days after implementation of a RRT.

It should be noted that traditional clinician led escalation protocols continued to occur alongside the newly introduced patient and relative led escalation service, both of which may have influenced the measured clinical outcomes. Effects of clinician and relative led escalation on clinical outcomes were separated in one study. Here, Brady et al.^30^ found that 24% of 40% relative activated RRT resulted in the transfer of the patient to the ICU compared to 60% of 1156 clinician activated RRT. Although patient and relative activation less often resulted in ICU transfer compared to clinician activation, patients and relatives may have escalated a subset of deteriorating patients missed by health‐care professionals.^30^

#### 3.3.2 Non‐clinical outcomes

Measures of non‐clinical outcomes centred on the number of patient and relative activated RRT and their reasons for activating it. The majority of studies reported the number of patient and relative activated RRT to monitor the potential for the patient activation intervention to overwhelm available resources.^14^, ^25^, ^26^, ^27^, ^29^, ^30^, ^31^, ^32^ However, the average number of patient and relative activated RRT reported across studies was 23 over an average time period of 1.5 years. The number of activations reported in each study is context dependent as the service was implemented on a different number of wards for different lengths of time between studies. Brady et al.^30^ presented the number of patient and relative activated RRT as a percentage of all RRT activations at 2.9%, supporting findings that the number of activations does not pose a substantial burden to the RRT.

The reasons patients and relatives activated a RRT were often cited as appropriate, meeting the pre‐defined criteria for RRT activation. A small number of studies reported that some patient and relative activated RRT were considered problematic and demanding by health‐care staff.^25^ All studies made reference to communication breakdown between health‐care staff, patients and relatives as contributing towards the reason for some, if not all, patient and relative activations. Here, reasons for RRT activation were not suspected patient deterioration but instead included concerns about the patients’ plan of care, their medication and pain control, their dietary status and their discharge.^26^ Six of the included grey literature websites reported research findings, providing information on the number of patient and relative RRT activations and reasons for these activations. Consistent with academic findings, the grey literature stated that patients and relatives reasons for activating the RRT were genuine and appropriate.

### 3.4 Quality assessment

The overall quality of the studies was fairly low. QATSDD scores ranged from 16% to 57%, with an average score of 31%. Few studies explicitly stated the study aims or objectives, and no studies justified their sample size or methods of data collection. Few studies provided descriptions of the analytic process or justification for the chosen analysis approach. One study made reference to the use of theory when implementing patient and relative led escalation. Theory was not used in any study to underpin the design and content of patient and relative led escalation. Research settings, procedures for data collection and recruitment data were adequately described in most studies. Quality assessments are available from authors on request.

## 4 DISCUSSION

The current systematic review explores how patients and their relatives have been engaged in escalating in‐hospital clinical deterioration. Evidence investigating patient and relative led escalation does not appear to explore the involvement of patients and relatives in monitoring and detecting patient deterioration.

Patient and relative led escalation is proposed as an intervention to reduce preventable deterioration within the reviewed studies. However, few studies were designed to establish the clinical effectiveness of patent and relative led escalation. This may be because large samples of patients would be required to assess reductions in relatively rare events, for example cardiac arrests. Some studies that did employ clinical outcome measures were poorly designed in that the effects of patient and relative led escalation on clinical outcome measures were not isolated from the effects of clinician led escalation.^31^, ^32^ Thus, any reported changes in clinical outcome measures could not be attributed solely to patient and relative led escalation. It is entirely possible that when patient and relative led escalation is implemented, this may lead to increased vigilance and hence escalation amongst health‐care staff resulting in improved clinical outcomes. It should be acknowledged that when the effects of relative and clinician led escalations on transfer to higher level care were separated, some relative led escalations did result in transfer of the patient to the ICU.^30^

The majority of studies used non‐clinical outcome measures to investigate issues of feasibility and acceptability surrounding patient and relative led escalation, exploring its impact on health‐care staff and their available resources. This reflects the infancy of research in this area and is entirely appropriate. Evaluating an intervention in a large‐scale trial first requires confidence that it is acceptable to users and does not have associated unintended (negative) consequences. Studies exploring patient and relative satisfaction found that they had favourable opinions towards the service.^14^, ^29^, ^32^

Studies had a lack of theoretical underpinning making it difficult to gain insight into the active components of the interventions.^34^ The low number of patient and relative activated RRTs reported in the academic and grey literature was interpreted as positive findings, showing that resources did not become overwhelmed. However, this may reflect an unwillingness by patients and relatives to participate in a behaviour that might be perceived as challenging health‐care staff.^35^ It will be important for future studies to explore possible mediating variables between the implementation of a patient and relative led escalation system and the outcome measures used, to better understand the mechanisms for any identified relationships. It is being increasingly recognized that specifying theory of change for an intervention is important for both implementation and replicability.^36^

Communication failure between health‐care staff, patients and relatives was cited as a reason for patient and relative led escalation in all studies. The types of communication failure reported were unrelated to a patient's deteriorating clinical condition. Communication failures that prompted patient and relative led escalation related to issues that increased the possibility of patient safety events and negatively affected patient and family experience.^30^ This finding is consistent with previous research which found that clinician activated RRTs not only identify deteriorating patients, but they also identify previously unknown systems issues, adverse events and preventable adverse events.^37^, ^38^ Highlighting previously unknown communication issues was a valuable unintended outcome of patient and relative led escalation. Indeed, accessing help from health‐care professionals who are independent from the ward/unit caring for the patient may be a vital function of this intervention. However, it could be argued that activating a RRT may not be the most appropriate or cost‐effective method of resolving concerns that are non‐life‐threatening.

The current systematic review has highlighted that patient and relative led escalation systems implemented in the reviewed studies do not consider the extent to which patients and relatives can monitor changes in the patients’ clinical condition and detect if they are deteriorating. Yet, to improve the management of clinical patient deterioration in hospital, patient and relative led escalation depends wholly on patients and relatives’ ability to effectively detect patient deterioration. Little is known about their ability to recognize signs of the patients deteriorating condition.

Of the available literature, one study revealed that patients and relatives felt unable to actively contribute to the management of their acute illness as their ability to recognize changes in their clinical condition was limited.^39^ Patients stated that they used the presence of new symptoms to indicate that their clinical condition was worsening. However, even when new symptoms were present, some patients were unsure of their significance and often did not interpret this as an indication that their condition was deteriorating.^39^ In line with this finding, researchers have developed a patient education intervention aimed at enhancing the self‐efficacy of hospitalized patients to recognize and report symptoms of deteriorating conditions. It was found that participants who received the intervention had significantly higher self‐efficacy to recognize and report symptoms post‐intervention compared to controls.^40^

### 4.1 Review limitations

Despite an inclusive search strategy, only two web search engines were used to search for a proportion of the grey literature. It is possible that relevant grey literature articles were not identified if they were stored on other databases that were not searched. The evidence included in the review lacked detail. Poor reporting may have resulted in an unduly negative assessment of the evidence. It is important that future research in this area is of high quality and is reported in sufficient detail so that methods can be replicated and refined.

### 4.2 Implications and recommendations

Patients and their relatives are likely to possess unique expertise on the patients’ status. Intuitively, it makes sense for patients and relatives to contribute towards the management of the deteriorating patient. However, in a complex organization, it is difficult to engage patients and relatives in a way that is feasible and acceptable, to allow the expertise of both patient and provider to be utilized. Patient and relative led escalation has been implemented in a number of hospitals despite a lack of empirical evidence to suggest that it is the most effective means of engaging patients and relatives to reduce preventable deterioration.

The reviewed evidence did not investigate the extent to which patients and relatives can effectively detect patient deterioration. The available research on this topic points to a need to improve patients’ and relatives’ ability to detect changes in the patients’ clinical condition indicative of deterioration. This warrants further investigation as it has important implications for the utility of patient and relative led escalation which rests on the assumption that patients and relatives can effectively detect clinical deterioration. Furthermore, patients and relatives often escalated patient care to resolve communication issues with health‐care staff that were unrelated to suspected clinical deterioration. It is recommended that health‐care organizations consider an alternative escalation route to allow patients and relatives to receive a timely response to concerns that are not life‐threatening, but relate to communication issues with health‐care staff.

## 5 CONCLUSIONS

Health‐care providers have leapt into involving patients and relatives in the management of patient deterioration. A more measured approach is now required to investigate the assumptions on which patient and relative led escalation is based. The reviewed evidence suggests that introducing patient and relative led escalation did not overwhelm staff and their available resources; however, it was difficult to establish the clinical effectiveness of the intervention. More high‐quality research and reporting is required to explore how the expertise of patients and relatives may be most effectively used, in conjunction with health‐care providers, to reduce preventable patient deterioration.

## SOURCE OF FUNDING

The University of Leeds, Leeds, West Yorkshire, UK, funded the PhD studentship through which this systematic review was conducted.

## CONFLICTS OF INTEREST

None.
