# Supplementary material for: Is there a role for patients and their relatives in escalating clinical deterioration in hospital? A systematic review
Source: Health Expect. 2016 Oct 26;20(5):818–25. doi: 10.1111/hex.12496 (PMC5600219; doi:10.1111/hex.12496)
Supplement: Supplementary file 2 [file HEX-20-818-s002.pdf]

Additional file 2

Academic and grey literature search strategies and results

**Database: Ovid MEDLINE(R) < 1990 to 2015 >**	
Search strategy:	
1	Patient* activat* (708)
2	Relative activat* (260)
3	Family activat* (209)
4	1 or 2 or 3 (1176)
5	Rapid response team* (568)
6	Medical emergency team* (337)
7	Critical care outreach team* (27)
8	Condition help (9)
9	5 or 6 or 7 or 8 (850)
10	4 and 9 (10)

**Database: PsycINFO < 1990 to 2015 >**	
Search strategy:	
1	Patient* activat* (293)
2	Relative activat* (98)
3	Family activat* (9)
4	1 or 2 or 3 (400)
5	Rapid response team* (23)
6	Medical emergency team* (24)
7	Critical care outreach team* (3)
8	Condition help (2)
9	5 or 6 or 7 or 8 (49)
10	4 and 9 (0)

**Database: CINAHL < 1990 TO 2015 >**	
Search strategy:	
1	Patient* activat* (1,935)
2	Relative activat* (168)
3	Family activat* (126)
4	1 or 2 or 3 (2,198)
5	Rapid response team* (385)
6	Medical emergency team* (250)
7	Critical care outreach team* (32)
8	Condition help (243)
9	5 or 6 or 7 or 8 (879)
10	4 and 9 (2,699)

**Database: Cochrane Library < 1990 to 2015 >**	
Search strategy:	
1	Patient* activat* (68)
2	Relative activat* (19)
3	Family activat* (2)
4	1 or 2 or 3 (88)
5	Patient* deteriorat* (124)
6	4 and 5 (1,076)

**Search engine: Google < 01/01/1990 to --/--/2015>**	
Search strategy:	
1	family rapid response team (6,480,000)
2	relative rapid response team (3,130,000)
3	patient rapid response team (18,700,000)
4	family medical emergency team (58,200,000)
5	relative medical emergency team (2,120,000)
6	patient medical emergency team (16,700,000)
7	family critical care outreach team (3,210,000)
8	relative critical care outreach team (533,000)
9	patient critical care outreach team (21,900,000)
10	family condition help (710,000,000)
11	relative condition help (388,000,000)
12	patient condition help (287,000,000)

**Search engine: Google Scholar < 01/01/1990 to --/--/2015>**	
Search strategy:	
1	family rapid response team (572,000)
2	relative rapid response team (712,000)
3	patient rapid response team (355,000)
4	family medical emergency team (596,000)
5	relative medical emergency team (337,000)
6	patient medical emergency team (969,000)
7	family critical care outreach team (90,300)
8	relative critical care outreach team (50,600)
9	patient critical care outreach team (54,200)
10	family condition help (3,210,000)
11	relative condition help (4,460,000)
12	patient condition help (3,030,000)
