# Supplementary material for: Is there a role for patients and their relatives in escalating clinical deterioration in hospital? A systematic review
Source: Health Expect. 2016 Oct 26;20(5):818–25. doi: 10.1111/hex.12496 (PMC5600219; doi:10.1111/hex.12496)
Supplement: Supplementary file 3 [file HEX-20-818-s003.pdf]

Additional file 3

Data extraction form for academic literature

Primary author and year:	Unique identifier:
Reviewer:	Date of extraction:

Topic	Instructions	Extracted data
Quality assessment	Refer to quality assessment form. Input overall score.	
Publication type	Select option	
Definition of patient/ family activated RRT.	Detail how patient/ family activated RRT is defined in the research.	
Study design	Detail the study design used e.g. qual, quant, cross-sectional, prospective …	
Sample	Who were participants? Eg. patients or clinicians. How were they sampled? Population sample drawn from, sample size, how calculated, representation, etc. Were characteristics accounted for (e.g. inc/ex criteria).	
Healthcare setting	Detail the specific setting researched: hospital, primary care, wards, teams etc.	
Location	Country and city.	
Context	Detail the context within which patient/ relative activated RRT is used; primary method, within an intervention etc.	
Study aims	Detail the aims, objectives and/ or hypotheses stated.	
Methods/ procedure	Detail the methods and procedure used to describe or evaluate	

	patient/ family activated RRT.	
Outcome measures	Detail outcome measures used.	
Timing	Length of time over which patient/ family activated RRT studied.	
Data analysis	Detail the data analysis strategy used.	
Key findings/ conclusions	Detail the key findings and conclusions drawn. Where applicable try to identify the effect of the patient/ family activated RRT component. How many patient/ family activated RRTs? Reasons? What were the outcomes for patients? What were the outcomes for healthcare staff?	
Strengths and weaknesses of the research	Detail any strengths and weaknesses of the research identified by the author.	
Generalisability	Author comments on generalizability of the research.	
Future research recommendations	Outline recommended future research/ actions relating to this specific topic which are detailed in the paper.	
Resource	Author and reviewer judgement of resources required for the research. Include resource needed for specific findings and feasibility for future applications.	
Additional notes	Enter as required	

Details of second review:

Second reviewer:	Date of extraction:
Second reviewer comments:	

Data extraction form for grey literature

Primary author and year or title of website:	Unique identifier:
Reviewer:	Date of extraction:

Topic	Instructions	Extracted data
Publication type	Select option	
Definition of patient/ family activated RRT.	Detail how patient/ family activated RRT is defined.	
Audience	Who is the target audience of the website? Is the website patient or staff facing or both?	
Host	Who is hosting the website? For example. Health organisation, private company, national body, charity etc.	
Resources provided	What resource does the website contain? Information, tools, research findings etc.	
Evaluation	Does the website contain any evaluation of the patient/ relative activated RRT?	

Second reviewer:	Date of extraction:
Second reviewer comments:	
