# Supplementary material for: Is there a role for patients and their relatives in escalating clinical deterioration in hospital? A systematic review
Source: Health Expect. 2016 Oct 26;20(5):818–25. doi: 10.1111/hex.12496 (PMC5600219; doi:10.1111/hex.12496)
Supplement: Supplementary file 4 [file HEX-20-818-s004.pdf]

Additional file 4

Characteristics of included academic studies

Lead author/ year	Paediatric or adult sample	Healthcare setting	Study design	Primary objective	Type of patient and relative led escalation	Main finding(s) relating to patient and relative led escalation
Bogert (2010)	Information not given	500 bed community hospital<br>Pilot ward- medical pulmonary unit	Descriptive design	Information not given	Activation of condition H team which has distinct staff from the RRT who triage care and determine whether RRT required	8 condition H activations in 13 weeks. All activations met at least one of the policy criteria.<br>No activations required RRT intervention.<br>Patients and relatives felt more empowered.<br>Most activations dealt with communication issues between patients, families and staff.<br>Some calls considered problematic and demanding by staff.
Brady (2014)	Paediatric patients	577 bed academic, freestanding secondary care children's hospital	Descriptive design, quantitative evaluation	To compare the rate of PICU transfer for relative versus clinician-activated RRT.<br>To compare relatives and clinicians reasons for activating RRT	Direct activation of the same RRT who respond to clinicians activations	83 relative activated RRT in 6 years (average of 1.2 MET activations per month).<br>Relative activations represented 2.9% of all RRT activations. Significant increase in relative activated RRT over study period.<br>24% of 40 relative activated RRTs resulted in transfer to PICU compared to 60% of 1,156 clinician activated RRTs.<br>Clinical deterioration more commonly the reason for clinician than relative activated RRTs. Relatives identified lack of response from clinicians and dismissive interaction between family and clinicians as reasons for activations. 37 family calls identified clinical deterioration.

Lead author/ year	Paediatric or adult sample	Setting	Design	Primary objective	Type of patent and relative led escalation	Main finding(s) relating to patient and relative led escalation
Dean (2008)	Paediatric patients	Children's teaching hospital	Descriptive design, qualitative evaluation	Information not given	Activation of condition H team which has distinct staff from the RRT who triage care and determine whether RRT required	42 condition H activations in 2 years.<br>15 activations due to management, coordination or plan of care.<br>9 activations due to medication and pain control.<br>6 activations due to discharge.<br>6 activations due to dietary status.<br>6 activations due to delays in service or amenities.<br>Communication breakdown between staff and patients was basis for all condition H activations.
Gerdik (2010)	Adult patients	696 bed adult level 1 trauma centre<br>Pilot wards- five medicine-surgery units and one oncology unit	Descriptive design, cross-sectional survey	Information not given	Direct activation of the same RRT who respond to clinicians activations	25 patient or relative activated RRTs in 2 years (48% of calls initiated by relatives, 52% by patient)<br>Patient/ relative activated calls were appropriate (no overload of false positives).<br>Reasons for call included 'something is just not right', worried, shortness of breath and increased pain.<br>Found significant increase in transfer to higher level care, a non-sig decrease in non-ICU AEs found and a significant decrease in mortality.<br>Survey showed patients and families very satisfied with patient/ family activated RRT.
Greenhouse (2006)	Information not given	520 bed tertiary care hospital	Descriptive design, semi-structured interviews	Information not given	Activation of condition H team which has distinct staff from the RRT who triage care and determine whether RRT required	21 condition H activations in 9 months.<br>Majority of calls met at least one of the two criteria.<br>Most calls related to communication issues between patients and clinicians.<br>Five of the calls were related to needing more effective pain management. Four were made by mistake. One was made due to chest pains.

Lead author/ year	Paediatric or adult sample	Setting	Design	Primary objective	Type of patient and relative led escalation	Main finding(s) relating to patient and relative led escalation
Hueckel (2012)	Paediatric patients	186 bed children's hospital<br>Pilot wards- Paediatric Bone Marrow Transplant Unit and Intermediate Care Unit.	Descriptive design, quantitative evaluation	To increase nursing and family awareness about the condition H service using formalised scripted teaching at the time of admissions.	Activation of condition H team which has distinct staff from the RRT who triage care and determine whether RRT required	47 RRT activations during the 12 week pilot and 2 of these were relative initiated condition H calls.<br>No significant difference in compliance with nurse education about condition H between the 2 pilot units.<br>PBMTU- monthly checks showed 64% to 90% (80% mean) of 38 eligible families received condition H teaching. 88% of 32 eligible families completed the family understanding survey. All but one family indicated that they had heard about condition H and could give a reason for calling.<br>Intermediate care unit- 107 of 159 admitted families received condition H teaching (from 53% to 85% of families each week). 81% of families participated in the survey. 98% of families had heard about condition H, 74% could describe a reason for calling condition H and 76% knew how to activate a condition H.
McCawley (2013)	Information not given	86-bed community hospital<br>Pilot wards- general surgery, medicine oncology, orthopaedics and progressive care	Descriptive design	To revise the condition H education program for staff, patients and families.<br>To observe staff members approach to teaching condition H protocols, patient and family knowledge about condition H and patient outcomes after improved condition H knowledge and usage.	Activation of condition H team which has distinct staff from the RRT who triage care and determine whether RRT required	91.7% of families received condition H education at pre-intervention which significantly increased to 97% post-intervention.<br>At post-intervention 481 family surveys were completed. Family understanding of when and how to call condition H was 80% overall.<br>There was a non-significant increase from 3 to 5 in number of condition H calls made from 3 months pre-intervention to 3 months post-intervention.

Lead author/ year	Paediatric or adult sample	Healthcare Setting	Design	Primary objective	Type of patient and relative led escalation	Main finding(s) relating to patient and relative led escalation
Odell (2010)	147 adult patients transferred to general hospital wards from the ICU (phase 1) and adult patients on two surgical wards who had been admitted from any ward (phase 2).	800 bed district general NHS hospital	Descriptive design	To introduce and evaluate a system that allowed patients and relatives to directly access RRT team through a process of self-referral.	Activation of Call 4 Concern team (UK name for condition H team) which has distinct staff from the RRT who triage care and determine whether RRT required	Phase 1: 12 C4C activations in 6 months.<br>Majority calls made by relatives. 2 cases where relative called C4C and patient was critically ill. In the other 10 cases, less critical intervention was needed.<br>Patient feedback questionnaires showed majority (n =25) felt they had enough information about C4C (83%) and felt reassured this service was available (90%)<br>Context assessment index showed that CCO and surgical ward staff felt the C4C project was being implemented in an environment receptive to change and conductive for person centred practice.<br>Phase 2: 27 C4C activations in 3 months.<br>85.7% of ICU staff had heard about C4C and 18.4% had been involved in explaining it to patients. Just over half of surgical ward staff had heard about C4C.
Ray (2009)	Paediatric patients	140-bed children's hospital	Descriptive design, cross-sectional survey	Information not given	Direct activation of the same RRT who respond to clinicians activations	Since family activated was introduced, mean number of RRT calls significantly increased from 16 to 24 calls per 1,000 discharges.<br>In 5% of all calls family concern was noted as reason for activation.<br>2 relative activated RRTs in a year.<br>Median number of calendar days between cardiac arrests increased from 34 to 104 days since initial implementation of RRT. Did not have sufficient data to evaluate impact of family activation on cardiac arrests.

Characteristics of included grey literature websites

Lead author, year and/or website title	Location	Audience	Host	Type of resource	Resource provided	Evaluation	URL
Applying patient and family centred concepts to rapid response teams	USA	Healthcare staff	Public-private partnership	Guidance	Guidelines and advice for healthcare staff on implementing patient and relative activation of RRT within the context of partnership and collaboration with patients and families.	No evaluation	http://www.macoalition.org/documents/pfac/RRT-family%20activation-IFCC-1%202%2009final.pdf
Bartoo (2009)	USA	Patients, relatives and healthcare staff	Healthcare organisation	General information and research findings	Information about the implementation of relative activated RRT at a hospital, justification for its implementation, how patients were educated about it, healthcare staff concerns about it and how the system has been used since its implementation.	Found that only one of the 6 RRT calls made by family during the pilot study were non-emergent.	http://www.mc.vanderbilt.edu:8080/reporter/index.html?ID=7703
Critical care outreach team: Patient and family access	USA	Patients and relatives	Healthcare organisation	General information and research findings	Power point presentation detailing the history of RRT at a hospital, outcomes after implementing RRT, plans for implementing patient and relative activated RRT including education, implementation and ongoing evaluation, results of pilot study and changes made based on pilot findings.	No patient and relative requests made for the activation of RRT during the pilot study	http://www.google.com/url?sa=t&rct=j&q=&esrc=s&source=web&cd=26&cad=rja&uact=8&ved=0CD8QFjAFOBQ&url=http%3A%2F%2Fwww.marylandpatientsafety.org%2Fhtml%2Fcollaboratives%2Fcondition_h%2Ftoolkit%2Fdocuments%2FLessons_Learned%2FCondition_Help_CARROLL_HOSPITAL.pdf&ei=wYH0VInzA4TP7Qav_4HwDw&usg=AFQjCNHgSRNdc08R83B-HxDoiTVDrHM8Bg&sig2=AcCsSEqNNCkFnuHHKbyzgw&bvm=bv.87269000,d.ZGU
Condition H (St. Joseph hospital)	USA	Patients and relatives	Healthcare organisation	Leaflet	Information in a leaflet about what condition H is, why the hospital is offering the service and when and how patients and relatives can activate the	No evaluation	http://www.google.com/url?sa=t&rct=j&q=&esrc=s&source=web&cd=98&cad=rja&uact=

					medical emergency team by calling a condition H		8&ved=0CEoQFjAHOFo&url=http%3A%2F%2Fwww.sjo.org%2FFor-Patients%2FAbout-Your-Hospital-Stay%2FCondition-H.aspx&ei=T1z0VL6YM4vgauvngJgH&usg=AFQjCNE0F8YvQXAuiEzYjeGvQJetlQ88Vg&sig2=WBoY0DYErOhsoQkHUlifwA&bvm=bv.87269000,d.d2s
Condition H (UPMC Shadyside)	USA	Patients and relatives	Healthcare organisation	Leaflet	A leaflet detailing the Josie King story, an example of when a patient deterioration went unrecognised which prompted the development and implementation of condition H, information about what condition H is, along with when and how to initiate it.	No evaluation	https://www.google.com/url?sa=t&rct=j&q=&esrc=s&source=web&cd=48&cad=rja&uact=8&ved=0CEwQFjAHOCg&url=https%3A%2F%2Fwww.patientsafetygroup.org%2Fuploads%2Fprojects%2F162%2Fwallsigndraft2.doc&ei=bZn1VMX2B4nX7Qa77IEQ&usg=AFQjCNFDOXiYULz3ta0UhcDdQHEj-d77RA&sig2=wlbPLFLfRFupeOOkrltFsg&bvm=bv.87269000,d.ZGU
Condition H brochure (UPMC Shadyside)	USA	Patients and relatives	Healthcare organisation	Leaflet	A leaflet detailing the Josie King story, information about what condition H is, along with when and how to initiate it.	No evaluation	http://www.josieking.org/uploads/WordDocs/UPMC_Shadyside_Condition_H_Brochure.pdf
Condition H brochure (UPMC Shadyside)	USA	Patients and relatives	Healthcare organisation	Leaflet	A leaflet detailing the Josie King story, information about what condition H is, along with when and how to use it and what happens when the RRT is activated using condition H	No evaluation	http://www.upmc.com/about/why-upmc/quality/excellence-in-patient-care/Documents/condition-help-brochure.pdf

Condition H (Help) (St. Mary's Health System)	USA	Patients and relatives	Healthcare organisation	General information	Information about when patients and most likely to experience a change in their condition and how the patient or relative can activate the RRT.	No evaluation	http://www.stmarysmaine.com/Patients-and-Visitors/condition-h-help.html
Condition Help (UPMC Shadyside)	USA	Patients and relatives	Healthcare organisation	General information	Information about what condition help is, why the hospital has condition help, how to call the RRT using condition help and what happens after the RRT has been called	No evaluation	http://www.google.com/url?sa=t&rct=j&q=&esrc=s&source=web&cd=3&cad=rja&uact=8&ved=0CDEQFjAC&url=http%3A%2F%2Fwww.upmc.com%2Fabout%2Fwhy-upmc%2Fquality%2Fexcellence-in-patient-care%2FPages%2Fcondition-h.aspx&ei=r5X1VLmQIsW67gaX2oH4Dw&usg=AFQjCNFR8QSv-B-z8CyeF-WWsbOwmxuwkA&sig2=k81V7GMdPiG2QsrRw78TPA&bvm=bv.87269000,d.ZGU
Condition Help (Children's hospital of Pittsburgh of UPMC)	USA	Patients and relatives	Healthcare organisation	General information	Information about what condition H is, how to activate the RRT using condition H, when to activate the RRT and when not to activate it	No evaluation	http://www.google.com/url?sa=t&rct=j&q=&esrc=s&source=web&cd=4&cad=rja&uact=8&ved=0CDcQFjAD&url=http%3A%2F%2Fwww.chp.edu%2FCHP%2Fcondition%2Bhelp&ei=r5X1VLmQIsW67gaX2oH4Dw&usg=AFQjCNHXhGj8ouSa1CdKRbNuZruQ3kaKEg&sig2=QTJrHXVAa_NQSPDvmgUIVg&bvm=bv.87269000,d.ZGU
Condition H(elp) brochure for patients and families (Institute	USA	Patients and relatives	Independent not-for-profit organisation	Leaflet	Information in a leaflet about what the RRT is and when and how to call the RRT.	No evaluation	http://www.google.com/url?sa=t&rct=j&q=&esrc=s&source=web&cd=2&cad=rja&uact=8

for healthcare improvement)							&ved=0CCoQFjAB&url=http%3A%2F%2Fwww.ihi.org%2Fresources%2FPages%2FTools%2FConditionHBrochureforPatientsandFamilies.aspx&ei=r5X1VLmQIsW67gaX2oH4Dw&usg=AFQjCNFWySI5aKphrQf7k2vvQwfRsZfIrw&sig2=VNlW5aHsSGJhNEYHCFr4bQ&bvm=bv.87269000,d.ZGU
Condition Help (H) toolkit	USA	Healthcare staff	Healthcare organisation	Guidance	Tools and resources are provided to aid hospitals with implementing condition H.	No evaluation	http://www.marylandpatientsafety.org/html/collaboratives/condition_h/Toolkit/#
Ehrig (2013)	USA	Healthcare staff	Magazine	General information	Information about the introduction of relative initiated rapid response at a hospital including why it was introduced, how it was implemented and the benefits of it.	No evaluation	http://www.americannursetoday.com/family-initiated-rapid-response-team/
Escalating care and the medical emergency team	USA	Patients and relatives	Healthcare organisation	Leaflet	Information in a leaflet about what the RRT is and when and how to call the RRT.	No evaluation	http://www.google.com/url?sa=t&rct=j&q=&esrc=s&source=web&cd=11&cad=rja&uact=8&ved=0CB4QFjAAOAo&url=http%3A%2F%2Fwww.geelongprivatehospital.com.au%2Findex.php%2Fdownload_file%2Fview%2F201%2F&ei=uVn0VPvxFoy27gbrs4HoDw&usg=AFQjCNFj8e737mn3a4E5gGbm7-W0h7tHvw&sig2=I33YKSr5Z0VJNdnemxi87A&bvm=bv.87269000,d.ZGU
Family activated rapid response	USA	Patients and relatives	Healthcare organisation	Leaflet	Information in a leaflet about what the RRT is and when and how to call the RRT.	No evaluation	http://www.google.com/url?sa=t&rct=j&q=&esrc=s&source=web&cd=75&cad=rja&uact=

							8&ved=0CDYQFjAEOEY&url=http%3A%2F%2Fwww.virginia.edu%2Fuvaprint%2FHSC%2Fpdf%2F90065.pdf&ei=xEv0VNOUGZPuaPfxgtAJ&usg=AFQjCNFLVOj_uSSzYvqoCaQgHgV4XC43oQ&sig2=3rYdA4_1cdLBFn4RP9drnw&bvm=bv.87269000,d.d2s
Family activation: The next generation of rapid response	USA	Healthcare staff	Magazine	Guidance	Information about dealing with concerns of staff and staff resistance to patient and relative activated RRT and further guidance on how to successfully implement it.	No evaluation	http://www.strategiesfornursemanagers.com/ce_detail/203768.cfm
Flow chart for Rapid Response Team Initiated by the Patient or Family Member/Visitor	USA	Healthcare staff	Healthcare organisation	Flow chart	Flow chart showing the different sequence of events that occur when a patient or relative activates the RRT. Information appears targeted towards informing nurses of what actions to take.	No evaluation	http://www.google.com/url?sa=t&rct=j&q=&esrc=s&source=web&cd=25&cad=rja&uact=8&ved=0CDkQFjAEOBQ&url=http%3A%2F%2Fwww.marylandpatientsafety.org%2Fhtml%2hFcollaboratives%2Fcondition_h%2Ftoolkit%2Fdocuments%2FImplementation_Planning_Tools%2FPtFm_Activated_RRT_Flowchart-UCH.ppt&ei=jkj0VPuXGfGu7Abv9YAY&usg=AFQjCNG5f7YBa8gBGgbik_jmAeZsRi1ONA&sig2=Et1GKaUsAxN-FrFkvqS-Kw&bvm=bv.87269000,d.ZGU
How patients and family members use the RRT	USA	Patients and relatives	Healthcare organisation	Leaflet	Information in a leaflet about what the RRT is and when and how to activate the RRT	No evaluation	http://www.google.com/url?sa=t&rct=j&q=&esrc=s&source=web&cd=68&cad=rja&uact=8&ved=0CEsQFjAHODw&url=http%3A%2F%2Fwww.tamc.a

							medd.army.mil%2Fmchk-dm%2Frapid_response_team%2Fdocs%2FTripler%2520RRT%2520Family%2520Brochure%2520final%2520ver.pdf&ei=TUv0VNC4GobkaPaXgugJ&usg=AFQjCNEBmUQXGsoUakpnWIgFt9pswzwD4g&sig2=9d6XXIh2H7XejqWKkUaF8w&bvm=bv.87269000,d.d2s
Implementation action planning document	USA	Healthcare staff	Healthcare organisation	Action planning tool	Condition H implementation action planning tool which instructs healthcare staff to list 3 actions their team will commit to in order to promote or advance implementation of the condition H program at their facility within the next week, month and 3 months.	No evaluation.	http://www.google.com/url?sa=t&rct=j&q=&esrc=s&source=web&cd=2&ved=0CCQQFjAB&url=http%3A%2F%2Fwww.marylandpatientsafety.org%2Fhtml%2Fcollaboratives%2Fcondition_h%2Ftoolkit%2Fdocuments%2FImplementation_Planning_Tools%2FConditionH_ActionPlanningDocument.doc&ei=XkjrVK2FLYi17gaPjICgBA&usg=AFQjCNFanWuOAXl0MQRk9_F-V76_dEc6Eg&sig2=g7Rev610RdZyHT7tDBhF9Q&bvm=bv.86475890,d.ZGU
Landro (2009)	USA	Patients, relatives and healthcare staff	Private newspaper company	Research findings	Research findings from the implementation of a patient and relative activated RRT in a children's hospital. Patients and relatives could directly call the RRT using the same system at hospital staff.	After a year, found "family concern" was behind 20% of the calls. More than half the patients in those calls had to be transferred to an intensive-care unit.<br>Mean number of RRT calls has increased significantly, to 24 calls per 1,000 discharges from 16. About two calls per	http://www.google.com/url?sa=t&rct=j&q=&esrc=s&source=web&cd=28&cad=rja&uact=8&ved=0CEkQFjAHOBQ&url=http%3A%2F%2Fblogs.wsj.com%2Fhealth%2F2009%2F08%2F31%2Fcalling-for-rapid-response-hospital-help-

						year have been placed by family members and both have required transfer to the ICU.<br>Most families prefer to have a professional call on their behalf. Family concern continues to be cited by staff as a reason for 6% of all their own calls to RRT.	should-be-family-affair%2F&ei=jkj0VPuXGfGu7Abv9YAY&usg=AFQjCNGNrNxa9bIEv2bdsuScLDBZtYZGtA&sig2=sWXLPF11NgM5BLerTVAozw&bvm=bv.87269000,d.ZGU
LaVelle (2011)	USA	Healthcare staff	Non-profit medical organisation	Guidance and research findings	Information and considerations to guide healthcare organisations in designing a patient and relative activated RRT to be implemented in their hospital and research investigating hospitals regarding their implementation of a patient and relative activated RRT.	Only 12.5% of the hospitals assessed incorporated patient and family activation into their initial rapid response systems; 58% delayed patient or family activation three or four years until their basic RRT program was running well.<br>Direct activation of the RRT by the patient or family was allowed or encouraged in 73% of the hospitals surveyed. 17% of hospitals chose an indirect approach where patients and families may initiate the request for assistance, but activation of RRT was limited to staff members<br>The volume of patient or family RRT calls generally ranges from zero to 24 calls per year. In one hospital, 48% of the 25 non-staff calls during the first two years were initiated by a family member and 52% were initiated by the patient.	http://www.sccm.org/Communications/Critical-Connections/Archives/Pages/Patient-and-Family-Activation-of-Rapid-Response-Teams.aspx
Information for patients and their carers	UK	Patients and relatives	Healthcare organisation	Leaflet	Information in a leaflet about what the RRT is, who can call them and when and how to call them.	No evaluation	http://www.google.com/url?sa=t&rct=j&q=&esrc=s&source

							=web&cd=53&cad=rja&uact=8&ved=0CC8QFjACODI&url=http%3A%2F%2Fwww.resourcesorg.co.uk%2Fassets%2Fpdfs%2FThe%252024-7%2520rapid%2520response%2520team.pdf&ei=r0_0VKqjOMmWarWMgNAK&usg=AFQjCNHBloyMOP9GMjMuZ8I_DgWV0zx6tw&sig2=TRsLvr4NNmqrpD2eBXe-FA&bvm=bv.87269000,d.d2s
Medical emergency teams: The Cleveland model	USA	Healthcare staff, patients and relatives	Healthcare organisation	Abstract	Does introducing a RRT reduce the volume of code blue events and improve patient survival, using a database of comparable hospital systems to compare with.	After introducing a rapid response system which could be activated by clinicians and by patients and relatives, there was a reduction in code blue events.	http://www.clevelandclinicmeded.com/live/courses/2011/quality11/abstracts/25-30-MarThomas-Tallman-DO.pdf
New rapid response team stress family involvement	USA	Healthcare staff	Magazine	Guidance	Guidelines and advice for healthcare staff on why they should implement a patient and relative activated RRT and how they could do it successfully	No evaluation	http://www.healthleadersmedia.com/content/HOM-223394/CE-Article-New-rapid-response-teams-stress-family-involvement
Pace in practice: A two-step process	USA	Patients and relatives	Healthcare organisation	Leaflet	Information in a leaflet about what the RRT is, who can call them and when and how to call them.	No evaluation	http://www.google.com/url?sa=t&rct=j&q=&esrc=s&source=web&cd=49&cad=rja&uact=8&ved=0CFUQFjAIOCg&url=http%3A%2F%2Fwww.barwonhealth.org.au%2Fservices%3Ftask%3Dcallelement%26format%3Draw%26item_id%3D162%26element%3D0e75c24e-de51-4b1a-a503-c9260a15dbdb%26method%3

							Ddownload&ei=DFv0VPaiEtSu7Aa7m4AI&usg=AFQjCNHZCo98KOAARdNp5zzHHeEIE3T30Q&sig2=qObZnMLZuhZFdT0TW-v_lg&bvm=bv.87269000,d.ZGU
Paediatric rapid response team	USA	Patients and relatives	Healthcare organisation	Leaflet	Information in a leaflet about what the RRT is, who can call them and when and how to call them.	No evaluation	https://www.med.unc.edu/cce/files/prrs/Peds%20RRT%20Brochure.pdf
Rabin (2013)	USA	Patients and relatives	Private newspaper company	General information	Information about staying safe in hospital including a description of condition H. Calling a condition H in hospital is compared to calling 911 at home.	No evaluation	http://khn.org/news/sidebar-coordination-of-care/
Rapid response team	USA	Patients and relatives	Healthcare organisation	General information	Information on a healthcare organisation website about what the RRT is and when and how patients and relatives can call the RRT.	No evaluation	http://www.lutheranhealthcare.org/Main/RapidResponseTeam.aspx
Rapid response team	USA	Patients and relatives	Healthcare organisation	General information	Information on a healthcare organisation website about what the RRT is and when and how patients and relatives can call the RRT.	No evaluation	http://www.dignityhealth.org/stmarymedical/patients-and-visitors/patients/rapid-response-team
Rapid response teams	USA	Patients and relatives	Non-profit organisation	General information	Information on how RRT operate and the benefits of having rapid response systems. Research findings from a survey.	Results of survey found all 34 hospitals had implemented RRT. Mean number of activations of RRT in 2009 was 114.7 21 Hospitals allowed patients and relatives to activate RRT Patients educated about the service using posters and brochures.	http://www.google.com/url?sa=t&rct=j&q=&esrc=s&source=web&cd=31&cad=rja&uact=8&ved=0CB4QFjAAOB4&url=http%3A%2F%2Fwww.mchky.org%2Fgetpage.php%3Fname%3Drapid_response_team&ei=GFT0VKf9CYTqaI-lgZgI&usg=AFQjCNGbHvmFnsnV-

							HiejrdlcN8XrHjp6A&sig2=0BH2NTdTqXM3pJ5O7kp7oA&bvm=bv.87269000,d.d2s
REACH: Patient and family activated escalation of care	Australia	Healthcare staff	Healthcare organisation	Research findings	Power point presentation detailing research findings, specifically the results of a patient and family activated escalation intervention	Number of patient and family activated RRT in the hospitals.<br>Orange Health Service- 20 months, 5 calls<br>Calvary Mater Newcastle- 13 months, 0 calls<br>Dubbo Base Hospital- 11 months, 1 call<br>Bathurst Base Hospital- 8 months, 0 calls<br>The Children's Hospital Westmead- 8 months, 11 calls<br>Balmain Hospital- 8 months, 0 calls<br>Hornsby Hospital- 4 months, 0 calls<br>Royal North Shore Hospital- 4 months, 2 calls	http://www.google.com/url?sa=t&rct=j&q=&esrc=s&source=web&cd=48&cad=rja&uact=8&ved=0CE0QFjAHOCg&url=http%3A%2F%2Fwww.isqua.org%2Fdocs%2Fedinburgh-powerpoint-presentations-2013%2F1235-c12--karen-luxford-kilsyth-wed-reach-patient-and-family-activated-escalation-of-care.pdf%3Fsfvrsn%3D2&ei=6kn0VK6ENc3maonGgrgO&usg=AFQjCNEB8bUrYX3bXOiF_V0-1wPzU97ktw&sig2=FrHDduunPsVTUdCK7r64_w&bvm=bv.87269000,d.d2s
Simmons (2006)	USA	Patients and relatives	Magazine	General information and research findings	Information about what condition H is and why staff were initially concerned about its implementation. Research findings after condition H calls had been reviewed by staff.	After reviewing condition H calls it was reported that team actions averted potentially harmful events in 69 percent of the calls placed. Averted events include respiratory compromise, medication errors, skin breakdown and potential falls.	http://www.todayshospitalist.com/index.php?b=articles_read&cnt=79
Stollery's rapid response team gives power to parents	Canada	Patients, relatives and healthcare staff	Healthcare organisation	General information and research findings	Information about why a hospital has implemented a relative activated RRT, an example of a relative activated RRT which identified deterioration and resulted in transfer to higher level care.	The RRT was activated about 70 times since the relative activated RRT service was introduced (no indication of timeframe given).	http://www.google.com/url?sa=t&rct=j&q=&esrc=s&source=web&cd=21&cad=rja&uact=8&ved=0CB4QFjAAOBQ&url=http%3A%2F%2Fwww.alberta

							healthservices.ca%2F3248.asp&ei=jkj0VPuXGfGu7Abv9YAY&usg=AFQjCNGoLKye-TOH3_y1BgYsh087sjlA-Q&sig2=Lops-k_e_lqyrH9FUw37Lg&bvm=bv.87269000,d.ZGU
Valley children's healthcare- rapid response team	USA	Patients and relatives	Healthcare organisation	General information	Information about the patient and relative activated RRT at a hospital including how RRTs operate, how patients and relatives can activate them and benefits of having RRTs on patient outcomes.	No evaluation	http://www.google.com/url?sa=t&rct=j&q=&esrc=s&source=web&cd=17&cad=rja&uact=8&ved=0CD4QFjAGOAo&url=http%3A%2F%2Fwww.childrenscentralcal.org%2FServices%2Fclinical%2Frapidresponse%2FPages%2FDefault.aspx&ei=-kf0VLzxLM72aKmsgqgN&usg=AFQjCNEDRBdMUwwJuf9zpx-MvknTbIK2ww&sig2=sCVC6YopVz8L04nxWyjcJg&bvm=bv.87269000,d.d2s
Voice a patient concern with condition help	USA	Patients and relatives	Healthcare organisation	General information	Information in a leaflet about what the RRT is and when and how to activate the RRT	No evaluation	https://www.ridgeviewmedical.org/patients-visitors/patient-safety/voice-a-concern-with-condition-help

* One website has become unavailable and cannot be included in the table
